# Pinch-off of microfluidic droplets with oscillatory velocity of inner phase flow

**DOI:** 10.1038/srep31436

**Published:** 2016-08-11

**Authors:** Pingan Zhu, Xin Tang, Ye Tian, Liqiu Wang

**Affiliations:** 1 Department of Mechanical Engineering, the University of Hong Kong, Hong Kong; 2 HKU-Zhejiang Institute of Research and Innovation (HKU-ZIRI), 311300, Hangzhou, Zhejiang, China

## Abstract

When one liquid is introduced into another immiscible one, it ultimately fragments due to hydrodynamic instability. In contrast to neck pinch-off without external actuation, the viscous two-fluid system subjected to an oscillatory flow demonstrates higher efficiency in breaking fluid threads. However, the underlying dynamics of this process is less well understood. Here we show that the neck-thinning rate is accelerated by the amplitude of oscillation. By simply evaluating the momentum transfer from external actuation, we derive a dimensionless pre-factor to quantify the accelerated pinch-off. Our data ascribes the acceleration to the non-negligible inner fluid inertia, which neutralizes the inner phase viscous stress that retards the pinch-off. Moreover, we characterize an equivalent neck-thinning behavior between an actuated system and its unactuated counterpart with decreased viscosity ratio. Finally, we demonstrate that oscillation is capable of modulating satellite droplet formation by shifting the pinch-off location. Our study would be useful for manipulating fluids at microscale by external forcing.

In many natural systems, coordination between dynamic and geometrical parameters plays a crucial role in retaining the reliability and efficiency for system performance. For example, a beating heart expands and contracts periodically to pump blood through vessels^1^, thereby maintaining one’s life; most male frogs distend vocal sacs to intensify the calls, thereby attracting females and reinforcing the success in reproduction^2^. In principle, the reliability and efficiency usually arise from the periodic deformation in system configuration coordinated with dynamic parameters such as pressure gradient. Technologically, human beings benefit from this coordination in developing new techniques, for instance the invention of steam engine^3^ by which high-pressure steam expands to perform mechanical work. Recently, scientists also use the coordination strategy to control droplet generation by acoustically actuating^4^,^5^ or mechanically vibrating^6^,^7^,^8^ fluid systems. Subjected to an oscillatory flow, the bulbous drops pulsate with the rarefaction and compression of fluid pressure, followed by a sudden breakup due to a fast drainage in the fluid neck^8^. The resultant micro-droplets have various potential applications, ranging from material synthesis^9^, chemical reactions and mixing^10^ to biological assays^11^, drug encapsulation and delivery^12^ and cell cultures^13^. In these applications, highly-controllable droplet generation with high throughput is usually required. Note that the formation of satellite droplets is unfavourable in the interest of controllable generation of droplets, and the production frequency is often limited by neck thinning velocity. It is thus essential to have deep understanding toward the dynamics of droplet breakup to meet the demand of uniformity and high throughput.

The dynamics of Newtonian threads rupture without external forcing have been well established for both liquid-in-air and two-fluid systems^14^. In the vicinity of pinch-off, the neck profile is self-similar and the minimum neck radius *R*_*min*_ obeys scaling laws of 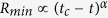, with exponent *α* determined by the asymptotic force balances under various circumstances^14^; *t*_*c*_ is the critical time at neck pinch-off and *t* is the time. Unlike pinch-off in air, viscous effect of the surrounding liquid is non-negligible in viscous two-fluid systems^15^. Stokes flow dominates two-fluid pinch-off when 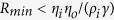
15, where *η*, *ρ* and *γ* represent respectively the dynamic viscosity, volumetric density and interfacial tension, and subscript *i* and *o* stand for inner and outer phase, respectively. In Stokes regime, capillary effect is counteracted by inner and outer viscous dissipations, while fluid inertia is negligible, which gives a linear scaling of *R*_*min*_ with *α* = 1^16^,^17^,^18^. The neck profile is featured by the asymmetric double-cone shape. The cone slopes only depend on the viscosity ratio *λ* (*λ* = *η*_*i*_/*η*_*o*_) of the two-fluid system^16^,^17^,^18^,^19^, independent of other parameters, such as nozzle diameter, interfacial tension, and density difference^18^. If the thinning neck radius can decrease down to the molecular scales, a transition to the thermal-fluctuation dominated pinch-off occurs when *R*_*min*_ < *L*_*T*_^20^,^21^. Here 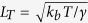 is the thermal length scale, with *k*_*b*_ and *T* being Boltzmann’s constant and temperature, respectively. The neck profile is symmetric and the liquid-liquid interface is rough due to the effects of thermal fluctuations. Both experimental^20^,^21^ and theoretical^22^ results suggest the power *α* ≈ 0.42 for thermal-fluctuation regime. However, *L*_*T*_ is usually nanometric for simple liquids, where the effects of thermal fluctuations are hardly to be observed at laboratory-scale. As such, Stokes flow is the final asymptotic regime in viscous two-fluid pinch-off, provided the molecular scales are not reached.

Except for the pinch-off of complex liquids in air^23^,^24^ and zero-viscosity fluids (for example bubbles) inside another highly viscous liquids^25^,^26^, the neck thinning dynamics of Newtonian fluids usually shows a universal behavior, which means that the neck profile and scaling law depends on neither initial nor boundary conditions. For instance, in liquid bridge experiments^27^, varying the stretching velocity can change the breakup locations^28^ and alter satellite droplet formation^29^, but the local dynamics of pinch-off remains unchanged and is identical to any pinch-off event involving that fluid^28^,^29^,^30^. However, in microfluidics, the channel confinement and dynamic process (such as flow rates) complicate the pinch-off scenario and would alter the neck scaling^31^,^32^,^33^,^34^ due to the alteration in local force balance. Utilization of external forcing introduces more variables to be considered. Previous experiments show that perturbing the inner fluid pressure displays an enhancement in droplet breakup^6^,^8^. Assuming the minimum neck radius *R*_*min*_ still obeys the scaling law 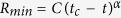 (*C* is the proportionality factor), the neck thinning velocity gives 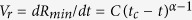. Influencing either the proportionality factor *C* or the exponent *α* would induce variations in the dynamics of droplet pinch-off, and thus the neck thinning velocity *V*_*r*_. Nevertheless, knowledge of pinch-off in microfluidics subjected to external forcing is limited, leaving several questions unaddressed like whether the external forcing would affect *C* or *α* or both and to what extent, and how it varies satellite droplet formation.

Here, we explore the dynamics of viscous two-fluid pinch-off in a co-flow microfluidic capillary device where the inner fluid velocity is oscillatory, actuated by mechanical perturbation (Fig. 1a). Our study lies into the Stokes flow regime, where the actuated neck radius still scales linearly with time prior to pinch-off (*α* = 1). However, the neck thinning velocity *V*_*r*_ is accelerated by the oscillation amplitude compared to that without oscillation (Fig. 1b–d). By estimating the pulsatile velocity of inner fluid at the ejection nozzle, we derive a dimensionless pre-factor to quantify the neck thinning velocity *V*_*r*_, and rescale the perturbed pinch-off. The pulsatile velocity reinforces the transient inner fluid inertia, which, in turn, counteracts inner viscous stress, thus enhancing the pinch-off. Moreover, we find that shifts in pinch-off location, raised from the oscillation amplitude, impact the formation of satellite droplets. Our work has significance in microscale hydrodynamic systems with external actuation, such as surface-acoustic-wave^5^ and mechanically-perturbed^6^,^7^,^8^ microfluidics.

## Materials and Methods

### Experimental procedure

**Figure 1:**
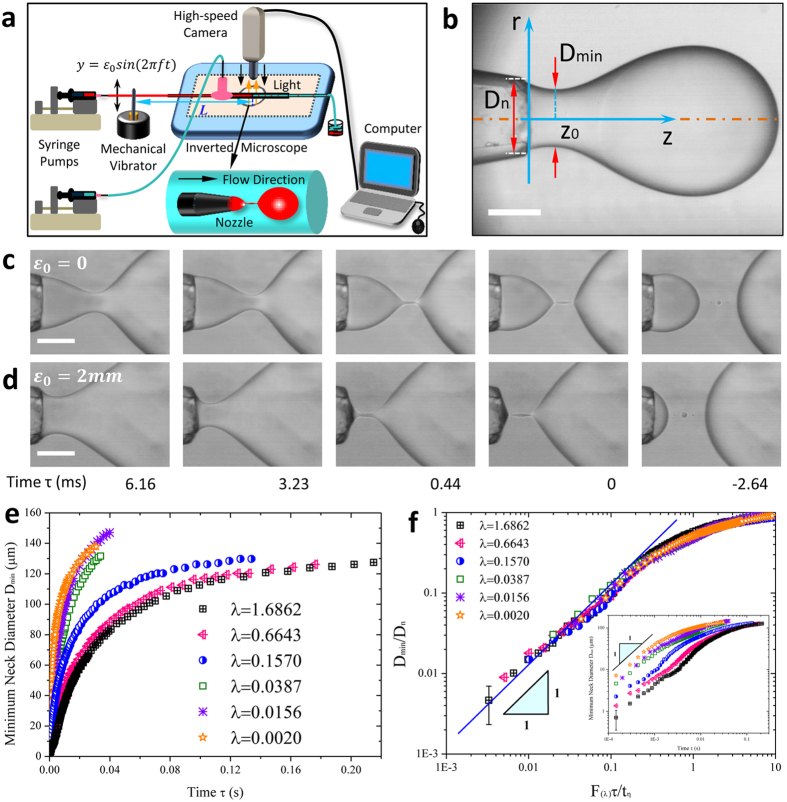
Neck pinch-off in microfluidics. (**a**) Experimental setup. (**b**) Image of a drop discharged from ejection nozzle. *D*_*n*_, *D*_*min*_ and *Z*_*0*_ are the nozzle diameter, minimum neck diameter and location of the minimum neck, respectively. Scale bar, 100 μm. (**c,d**) Snapshots of (**c**) unperturbed and (**d**) perturbed neck thinning *versus* time *τ* remaining to pinch-off (see supplementary Movies S1,S2,S3,S4,S5). In time period 0 < *τ *< 6.16 ms before pinch-off, the difference in neck diameter for perturbed case (*ε*_*0 *_= 2 mm) is larger than that for unperturbed case, showing the enhancement of pinch-off by mechanical perturbation. A suction of inner fluid towards the nozzle in (**d**) characterizes the last stage of neck pinch-off modulated by perturbation. The viscosity ratio of fluid system is *λ *= 0.157. Inner and outer fluid flow rates are *Q*_*i*_ = 0.3 mL h^−1^ and *Q*_*o*_ = 1 mL h^−1^, respectively. Scale bars, 100 μm. (**e**) Plot of minimum neck diameter *D*_*min*_ *versus* time *τ* for unperturbed two-fluid pinch-off with various viscosity ratios. Neck thins faster for fluid system with smaller viscosity ratio. (**f**) Scaling law for unperturbed neck pinch-off with different viscosity ratios. In Stokes regime, linear scaling gives 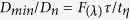. Inset: minimum neck diameter *D*_*min*_ *versus* time *τ* plotted in a log-log plane. The error comes from the limiting resolution in the captured image.

We conducted experiments in a co-flow capillary microfluidic device that was fabricated by aligning one round glass capillary with a taped nozzle (outer diameter of the nozzle *D*_*n*_ = 151.443 μm, Fig. 1a,b) inside another intact capillary (inner diameter 580 μm). Both inner and outer fluids were injected by syringe pumps (Longer Pump) into the microcapillary device, Fig. 1a. The outer fluid flow rate was kept as *Q*_*o*_ = 1 mL h^−1^, while inner flow rate *Q*_*i*_ was varied (0.02 mL h^−1^ ≤ *Q*_*i*_ ≤ 0.45 mL h^−1^). In the operation window of *Q*_*o*_ and *Q*_*i*_, only dripping was observed, no jetting occurred. A mechanical vibrator (Pasco Scientific, Model SF-9324) perturbed the inner fluid microtubing (inner-wall radius *R*_*iw*_ = 0.43 mm) in the gravitational direction with a displacement of 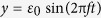. The perturbed microtubing was put nearly straight and meanwhile enabled to be displaced freely without deformation by the vibrator. The two ends of the microtubing were held still during experiments, one connected to the syringe needle and the other to the device (Fig. 1a). The distance *L* (Fig. 1a) between the perturbing location and nozzle was constant to be *L* = 40 cm. We studied the oscillation amplitude *ε*_*0*_ between 0 and 5 mm, with the upper bond limited by the vibrator, and examined frequency *f *≤ 100 Hz. When frequency is higher than 100 Hz, enhancement of pinch-off decays significantly^35^,^36^, probably due to two reasons: first, amplitude *ε*_*0*_ dissipates when frequency is too high; second, according to linear stability analysis^14^, when frequency is larger than a critical value, the cut-off wavenumber *kR*_*0*_ > 1 (*k* is the wavenumber and *R*_*0*_ is the unperturbed jet radius) and thus prevents the external perturbation from growing (see the Supplementary Information for validation). The flow was visualized, monitored and recorded (images and videos) by an inverted microscope (Nikon Eclipse TS100, Inverted Microscope) equipped with a high-speed camera (MotionPro^®^ X4, IDT, Taiwan). Captured images and videos were analyzed by *ImageJ*.

Water-in-oil two-phase flow was examined in experiments, where outer fluid was silicone oil (viscosity: *η*_*o*_ = 492.875 mPa s), and inner fluids were various glycerol-water mixtures (viscosity: 0.992 mPa s ≤ *η*_*i *_≤ 831.07 mPa s). This choice of two-fluid systems enables a wide range of viscosity ratios (10^−3^ < *λ *< 10), but narrow variations in interfacial tension (24.2 mN m^−1^ ≤ *γ* ≤ 32 mN m^−1^) and fluid density (0.998 g cm^−3^ ≤ *ρ*_*i*_ ≤ 1.261 g cm^−3^), so as to isolate the effect of viscosity ratio on pinch-off dynamics. Viscosity was measured by a viscometer (microVISC^TM^, RheoSense, Inc.), interfacial tension by a ring tensiometer (Surface Tensiometer 20, Cole-Parmer), and density by quantifying the volume of a known-mass fluid.

### Estimate of the inner fluid oscillatory velocity *V*_*n*_

With perturbation, the inner phase pressure is disturbed periodically, so that the velocity *V*_*n*_ of the inner fluid ejected from the nozzle pulsates with time. We have made a simple analysis to determine the pulsatile velocity *V*_*n*_. Before accounting for the effect of mechanical perturbation, we first consider the unperturbed case, in which the inner phase flow rate *Q*_*i*_ is estimated by Poiseuille’s law^37^,


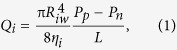


where *R*_*iw*_ is inner-wall radius of the microtubing (*R*_*iw*_ = 0.43 mm), *η*_*i*_ is the viscosity of inner fluid, *P*_*p*_ and *P*_*n*_ are pressures respectively at the perturbing location and the nozzle, and *L* is the length between the two locations. Based on *Q*_*i*_, we evaluate the steady ejection velocity of the inner fluid as,


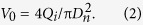


**Figure 2:**
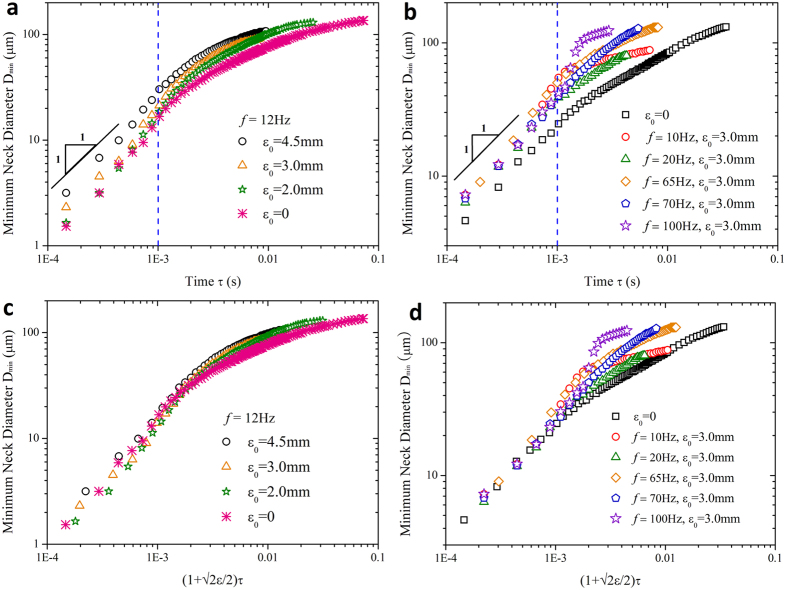
Acceleration of pinch-off with oscillatory velocity. (**a,b**) Effects of oscillation amplitude (**a**) and frequency (**b**) on pinch-off. Compared with unperturbed case, neck thins faster in the perturbed situation, but still displays linear thinning dynamics within the last ~1 ms (indicated by the dashed line) before pinch-off. The acceleration of pinch-off increases with oscillation amplitude in (**a**). By contrast, for different frequencies in (**b**), the enhancement of pinch-off shows weak dependence on the perturbation frequency. (**c,d**) Dynamics of perturbed neck thinning *versus* rescaled time for various oscillation amplitudes (**c**) and frequencies (**d**). Time *τ* is rescaled by multiplying a dimensionless pre-factor 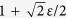. In (**a**,**c**), *Q*_*i*_ = 0.45 mL h^−1^, *Q*_*o*_ = 1 mL h^−1^; in (**b**,**d**), *Q*_*i*_ = 0.3 mL h^−1^, *Q*_*o*_ = 1 mL h^−1^. Viscosity ratio, *λ *= 0.0387.

For perturbed case, we simply assume that the gravitational displacement of 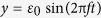 modifies the steady pressure *P*_*p*_ into the time-dependent *P*_*p*_(*t*),


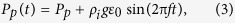


with inner fluid density being *ρ*_*i*_ and the gravitational acceleration being *g*. Since the linear pinching time-scale *t*_*l*_ (*t*_*l*_ ~ 1 ms, which is shown in the next section, Fig. 2) is much smaller than the perturbation period *t*_*p*_ (*t*_*p *_≥ 10 ms for *f* ≤ 100 Hz in experiments), we assume that the Poiseuille’s law is still valid to estimate the fluctuating inner flow rate *Q*_*i*_(*t*) in the linear pinch-off regime. When *P*_*p*_ is replaced by *P*_*p*_(*t*) in Eq. (1), we obtain *Q*_*i*_(*t*) as a function of time *t*,


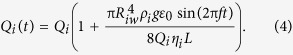


Now the oscillatory velocity *V*_*n*_ discharged from the nozzle is approximated to be 
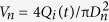
, and hence in the form of


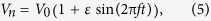


where 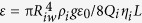 is the dimensionless amplitude of the fluctuation term.

Although we ignore several effects that might influence the transient velocity *V*_*n*_, such as shaking and deformation of the microtubing, and fluid acceleration in the microtubing, the simplified form of *Q*_*i*_(*t*) in Eq. (4) (as well as *V*_*n*_ in Eq. (5)) does characterize essentially how oscillation amplitude *ε*_*0*_, in association with material properties (*ρ*_*i*_ and *η*_*i*_) and geometrical parameters (*R*_*iw*_ and *L*), modulates the ejection velocity. We verify Eq. (4) by comparing the experimental measurements to theoretical predictions of the inner fluid discharged volume (see supplementary Figs S1 and S2). Moreover, we will show in the next section that pinch-off is enhanced by the vibration in the gravitational direction, while no enhancement is identified when the vibration is parallel to the horizontal plane (see supplementary Fig. S3). This difference suggests the dominant effect of gravity in impacting neck pinch-off. As a result, the above analysis paves a way to evaluate the average momentum transferred into the fluid system, to quantify the acceleration of pinch-off with perturbation, to rescale the perturbed neck pinch-off, and to account for the non-negligible inner fluid inertia in perturbed case, which are shown in the next section.

## Results and Discussion

### Scaling of classical viscous two-fluid pinch-off

We start from the case without oscillation but with the influence of viscosity ratio *λ*. Keeping silicone oil as the outer fluid, and choosing various glycerol-water mixtures as the inner fluid, we can tune viscosity ratio *λ* over three magnitudes (10^−3^ < *λ *< 10). Fluid system with smaller *λ* means less viscous inner fluid in the context, and *vice versa*. Figure 1e compares unperturbed minimum neck diameter *D*_*min*_ (Fig. 1b, *D*_*min*_ = 2*R*_*min*_) *versus* time *τ* (*τ* = *t*_*c*_ − *t*, time remaining to neck pinch-off) for different viscosity ratios *λ.* Fluid system with smaller viscosity ratio pinches off faster (Fig. 1e) due to the less inner viscous resistance. In the vicinity of pinch-off, a linear scaling of the neck diameter is presented (inset of Fig. 1f). In this linear pinch-off scenario, two-fluid Stokes flow dominates, where inner axial viscous stress 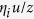 (with the kinematic estimate being 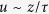, *z* the length scale of the pinching neck and *u* the axial velocity of fluid flowing out of the neck), outer radial viscous stress 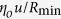, and capillary pressure 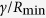 compete with each other^15^. This viscous-capillary force balance yields the scaling of neck diameter as 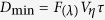 (see the Supplementary Information for derivations), where 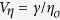 is viscous-capillary velocity, and *F*_(*λ*)_ is a dimensionless proportionality factor that can be evaluated from linear stability analysis^16^,^17^,^18^,^38^. Normalizing *D*_*min*_ by the nozzle diameter *D*_*n*_, we obtain a linear scaling of the minimum neck diameter in the form of


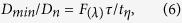


with viscous time scale 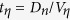. Based on the analysis by Tomotika^38^, we evaluate *F*_(*λ*)_ for different viscosity ratios (see the Supplementary Information for the estimate of F(λ)), and replot the data in Fig. 1f, which is in good agreement with previous study of viscous two-fluid drop pinch-off^15^,^16^,^17^,^18^.

### Perturbation accelerated pinch-off

Now we consider the fluid system with fixed viscosity ratio (*λ* = 0.0387) under periodic perturbation. When undergoing pinch-off subjected to an oscillatory flow, the droplet initially grows in its size until the neck appears. Afterwards, a sudden suction of the inner fluid, caused by the pulsatile velocity *V*_*n*_ (Eq. (5)), triggers and subsequently accelerates the neck pinch-off (exampled in Fig. 1d, and see supplementary Movies S1,S2,S3,S4,S5). The above process is identical to any individual pinch-off with oscillation (see supplementary Movie S6)). As a result, actuated neck thinning velocity *V*_*r*_ is faster than the case without actuation, while the linear neck thinning regime features both cases in the vicinity of pinch-off (Fig. 2a,b). The neck thinning velocity *V*_*r*_ increases apparently with the oscillation amplitude *ε*_*0*_ (Fig. 2a). However, the pinching velocity weakly depends on the oscillation frequency for *f *≤ 100 Hz (Fig. 2b), probably due to the mismatch between the two time scales, the linear pinching time scale *t*_*l*_ (*t*_*l*_ ~ 1 ms; see dashed line in Fig. 2a,b) and the perturbation period *t*_*p*_ (*t*_*p *_≥ 10 ms for *f *≤ 100 Hz).

We now quantify the acceleration of perturbed pinch-off by estimating the extra momentum contribution from mechanical vibration. After simply calculating the root mean square of the fluctuation term in Eq. (5), 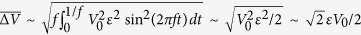, we extrapolate that the average momentum is increased by a ratio of 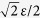 due to mechanical vibration, and hypothesize that the neck thinning velocity is accelerated by a ratio of 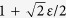 as an estimate. To verify this hypothesis, we replot Fig. 2a,b by rescaling *D*_*min*_ *versus* the product of time *τ* and the dimensionless factor 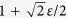. After rescaling, all data essentially collapse onto a single master curve in the vicinity of pinch-off (Fig. 2c,d). The enhancement of pinch-off comes thus from the additional momentum transportation induced by mechanical vibration, and is proportional to the dimensionless amplitude *ε*.

### Rescaling perturbed neck thinning with various viscosity ratios

**Figure 3:**
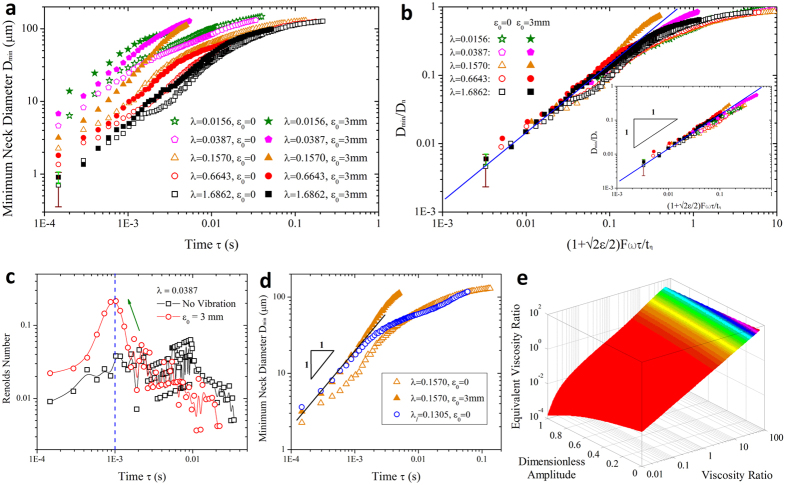
Influence of oscillatory flow on two-fluid pinch-off with various viscosity ratios. (**a**) Minimum neck diameter *versus* time for perturbed and unperturbed pinch-offs with various viscosity ratios. (**b**) Rescaling the perturbed neck pinch-off in linear pinching regime, 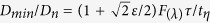. Inset: representative data in the linear scaling regime. (**c**) Comparison of inner fluid radial Reynolds numbers for perturbed and unperturbed cases. Perturbed Reynolds number *Re* (circle) increases sharply during the last stage of pinch-off, and thus surpasses unperturbed *Re* (square), indicating an enhancement of inner fluid inertia by perturbation. (**d**) An example of equivalent neck thinning behavior. Perturbed system (*λ* = 0.157, filled triangle) displays almost the same pinch-off dynamics as an unperturbed system with less viscous inner fluid (*λ*_*l*_ = 0.1305, circle) in the linear pinch-off regime. (**e**) Equivalent viscosity ratio *λ*_*l*_ as a function of dimensionless amplitude *ε* and viscosity ratio *λ* of the perturbed system. For the calculated domain, 0 ≤ *ε* ≤ 1, 10^−2^ ≤ *λ* ≤ 10^2^, and the resultant equivalent viscosity 10^−4^ < *λ*_*l*_ < 10^2^.

In subsequent tests, we focus on the influence of viscosity ratio on fluid systems with oscillation. In comparison to unactuated case, the oscillation-enhanced pinch-off has a faster neck thinning rate for all fluid systems examined, as shown in Fig. 3a. Note that Fig. 2b only indicates a dependence of neck thinning velocity on oscillation amplitude *ε*_*0*_, but Fig. 3a further clarifies the dependence on the dimensionless amplitude *ε*, in that larger enhancement of pinch-off is observed for fluid system with the same *ε*_*0*_ but smaller viscosity ratio *λ* (smaller *η*_*i*_ in experiments). Therefore, Eq. (6) fails in scaling the perturbed neck thinning with arbitrary oscillation amplitudes and viscosity ratios. From previous discussions, neck thinning velocity is accelerated by a ratio of 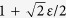. We then rescale the neck shrinkage behavior via multiplying the right hand side of Eq. (6) by the dimensionless pre-factor 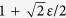:


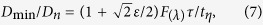


as shown in Fig. 3b (data rescaled from Fig. 3a). To highlight this rescaling, we replot representative data close to pinch-off in the inset of Fig. 3b, where all data falls onto a single master straight line with unit-slope, in excellent agreement with Eq. (7).

### Non-negligible inner fluid inertia

Having quantified the accelerated pinch-off, we now explore the origin of the acceleration in pinch-off with oscillatory flow. For conventional pinch-off scenario, two-fluid Stokes flow dominates near the singularity. However, oscillation modifies the force balance via disturbing the flow velocity, therefore affecting the whole pinch-off dynamics. A recent study proposed that, when pinching in air, fluid thread passes through a plethora of transient regimes before transferring into the final inertial-viscous regime^39^. Likewise, various regime transitions also exist in two-fluid pinch-off scenario before the final Stokes regime arrives, as indicated by the deviation of slope from unity when *τ* > 1 ms (Fig. 3a), especially for cases with high viscosity ratios (for example *λ *= 1.6862, open square in Fig. 3a). Oscillatory flow, however, smooths the original slope-varying curve into a more uniform unit-slope form (Fig. 3a, filled square for *λ* = 1.6862 with oscillation) by impacting the transient local force balance.

We hypothesize that oscillation amplifies the effects of axial inner fluid inertia. During pinch-off in Stokes regime, inner fluid thread of radius *R*_*min*_ and length *z* thins radially with velocity *V*_*r*_, while fluid flows axially out of the thread with velocity *u*. Local volume-flux conservation requires 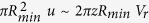. Provided 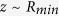
15, *u* is thus estimated as 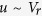. Since neck thinning velocity *V*_*r*_ is accelerated by the oscillation amplitude, *u* increases with *ε*, so does the inner axial fluid inertia 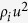. To verify this hypothesis, we compare the local axial Reynolds number *Re* in the neck for perturbed and unperturbed cases, respectively. Having 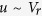 and 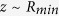 (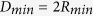), we assume that the axial 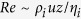 is of the same magnitude of the radial 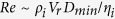. Figure 3c displays the results of radial *Re versus* time *τ* during neck pinching (*λ* = 0.0387). Close to pinch-off, a sharp increase of *Re* occurs for the perturbed neck, while undisturbed *Re* varies more gently. The increased *Re* indicates an undoubted increase of inner fluid inertia due to perturbation. It is now probably invalid to ignore the effects of oscillation-enhanced fluid inertia in determining the neck thinning dynamics. Actually, the increased fluid inertia neutralizes the inner fluid viscous dissipation.

We now elucidate the counterbalance between the enhanced inner fluid inertia and inner viscous stress. Attributed to oscillation-enhanced inner fluid inertia, the effective inner dissipation is weakened roughly to be 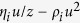 (compared to unperturbed case of 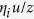). Because of the counterbalance, the perturbed pinch-off displays an equivalent neck thinning dynamics to the unperturbed case with smaller inner viscosity *η*_*i*_ (smaller viscosity ratio *λ* too). Preliminary, an observation (Fig. 3a) supports this speculation; for example, fluid system with viscosity ratio *λ* = 0.6643 disturbed by *ε*_0_ = 3 mm (filled circle, Fig. 3a) has the similar neck thinning dynamics to the unperturbed “less viscous” system with *λ* = 0.157 (open triangle, Fig. 3a).

**Figure 4:**
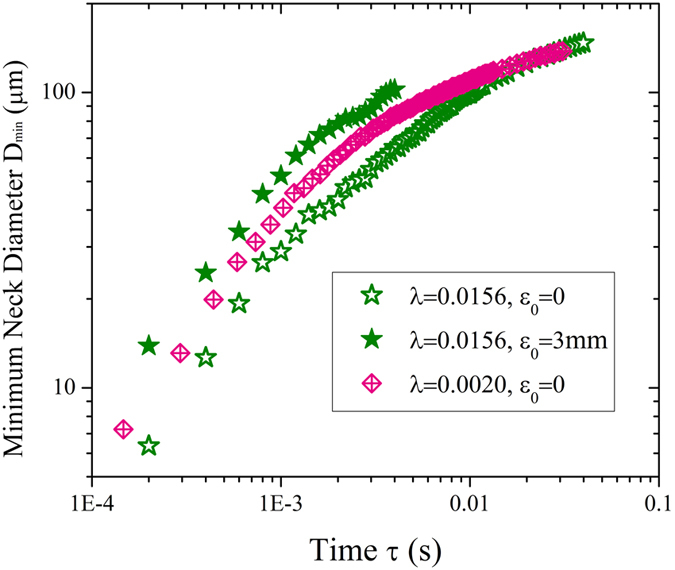
Dynamics of distilled water (crossed diamond) and 47 wt.% glycerol + 53 wt.% water (star) pinch-off in silicone oil. Although glycerol-water mixture pinches slower (open star) than pure water (crossed diamond), perturbing the mixture by appropriate amplitude (filled star) have the mixture pinch faster than water.

To quantify this “less viscous” performance due to perturbation, we explore the equivalent viscosity ratio *λ*_*l*_ corresponding to a perturbed system with viscosity ratio *λ* (*λ*_*l*_ < *λ*). This can be done by calculating 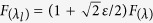 from Eq. (7). Take system with *λ* = 0.157 perturbed by *ε*_0_ = 3 mm for example, we find out *λ*_1_ = 0.1305. Experiments show that, close to pinch-off, the perturbed system with *λ* = 0.157 pinches equivalently to the undisturbed one with *λ*_*1*_ = 0.1305 (Fig. 3d). Furthermore, an equivalence between *λ*_*l*_ and *λ* with arbitrary *ε* is developed (Fig. 3e) based on the formula: 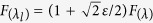. According to above analysis, we can even modulate a glycerol-water mixture undergoing “less viscous” pinch-off dynamics than distilled water by exerting appropriate oscillation amplitude (Fig. 4). The equivalence of pinch-off dynamics indicates potential in breaking highly viscous liquid in an easier manner by applying an oscillatory flow. Our findings show that dynamics of neck thinning in the linear regime is controlled by both fluid properties and oscillation amplitude. By characterizing the combined effects of viscosity ratio and dimensionless amplitude (Eq. (7) and Fig. 3e), we can predict the dynamics of linear neck pinch-off.

### Shifts of pinch-off location with perturbation

**Figure 5:**
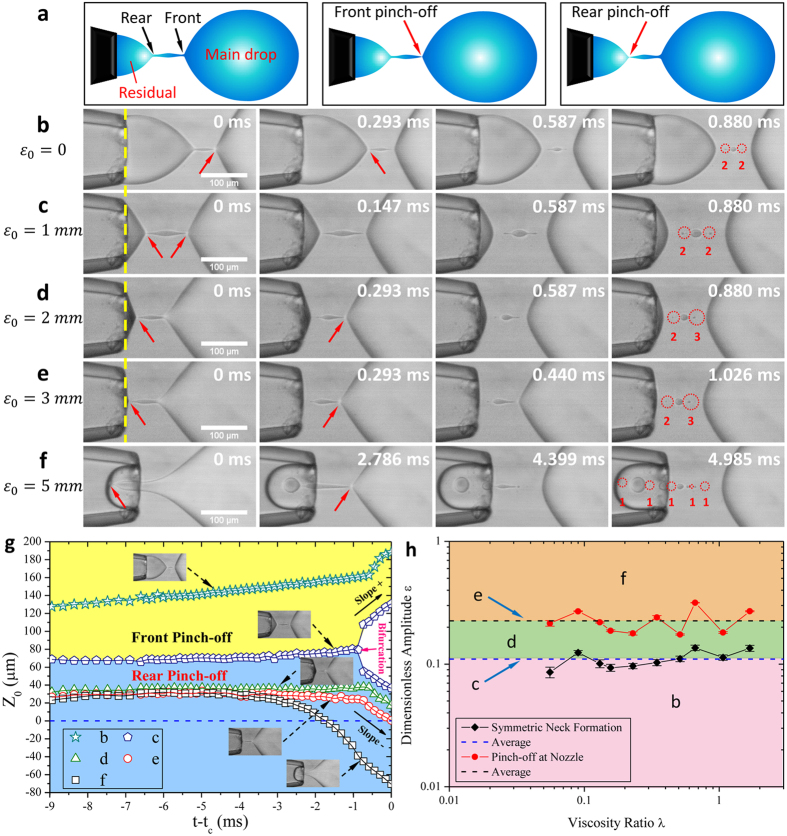
Shifts of pinch-off location with oscillation. (**a**) Schematic showing two possible pinch-off locations: front pinch-off and rear pinch-off. (**b–f**) Snapshots of pinch-off location transition and satellite droplet formation. In (**b–f**), the first and second rows display the first and second neck pinch-off (arrows), respectively; the third row shows an instant for satellite droplet formation; the last row presents the instant when all satellites are formed. The less visible satellites are indicated by circles and the numbers below. Time value on snapshots indicates the time after the first pinch-off. (**b**) *ε*_0_ = 0, front pinch-off occurring before rear pinch-off. (**c**) *ε*_0_ = 1 mm, symmetric neck formation. (**d–f**) Rear pinch-off occurring before front pinch-off. (**d**) *ε*_0_ = 2 mm, pinch-off outside the nozzle. (**e**) *ε*_0_ = 3 mm, pinch-off at the nozzle. (**f**) *ε*_0_ = 5 mm, pinch-off inside the nozzle. (**g**) Evolution of the location of minimum neck *Z*_*0*_ *versus* time (*t* − *t*_*c*_) during the pinch-off. *t*_*c*_ is the critical time for the first pinch-off. Data are measured from cases in (**b–f**). (**h**) Transition of pinch-off location with dimensionless amplitude *ε*. Regions (**b**,**d**,**f**) are separated by boundaries (**c**,**e**). *ε*_*c*_ = 0.110 for the symmetric neck formation (**c**); *ε*_c_ = 0.225 for pinch-off at the nozzle (**e**).

Besides neck thinning dynamics, pinch-off location also changes with applied oscillation. When breaking up, the liquid thread that connects the main drop and the residual fluid can pinch off either at its front or rear side^40^,^41^ (Fig. 5a, and see arrows in Fig. 5b–f). Compared with unactuated case (Fig. 5b), applied oscillation shifts pinch-off location upstream towards the nozzle (Fig. 5c–f, and see supplementary Movies S1,S2,S3,S4,S5) due to the pulsatile velocity *V*_*n*_. More remarkably, while front pinch-off occurs before rear pinch-off for unperturbed case (Fig. 5b), large driving amplitude *ε*_*0*_ can introduce the reversed case (Fig. 5d–f), between which a symmetric neck forms with front and rear pinch-offs occurring simultaneously (Fig. 5c). Meanwhile, oscillation can also induce a transition of pinch-off location from outside the nozzle (Fig. 5b–d) to inside the nozzle (Fig. 5f).

We map the pinch-off locations in Fig. 5g by tracing the location of minimum neck *Z*_*0*_ (Fig. 1b) *versus* time (*t* − *t*_*c*_) before pinch-off. Two boundaries are emphasized in Fig. 5g: symmetric neck formation (*Z*_*0*_ bifurcates, pentagon) and pinch-off at the nozzle (*Z*_*0*_ = *0* when *t* = *t*_*c*_, circle). The transition of pinch-off location is the result of momentum transport. Because axial velocity *u* of inner fluid scales as 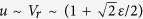, it is reasonable to assume that the two boundaries correspond to certain critical values of *ε*. Experimentally, we find that, in the tested range of viscosity ratios, *ε*_*c *_= 0.110 and *ε*_*c*_ = 0.225 (Fig. 5h) are the two critical values for the symmetric neck formation and pinch-off at the nozzle, respectively. These results provide a method to precisely manipulate pinch-off location by using oscillatory velocity.

Being susceptible to the details of breakup^40^,^41^, satellite droplet formation varies with the shift of pinch-off location. The multi-breakup of liquid thread generates multiple satellite and subsatellite droplets (circles in Fig. 5b–f) owing to the self-repeated neck formation^42^,^43^. With increased oscillation amplitude, the number of thread fragmentation rises, inducing more satellites and subsatellites (Fig. 5b–f); for instance, as many as ten satellites are generated with *ε*_*0 *_= 5 mm (Fig. 5f). In addition, the size distribution of satellite drops relies on the symmetry of neck formation. Symmetric pinch-off induces symmetric size distribution (Fig. 5c); asymmetrical neck formation otherwise renders the size distribution asymmetric (Fig. 5b,d,e). Astonishingly, rear pinch-off inside the nozzle produces satellite drops with descending size distribution (Fig. 5f), which are similar to those generated by the tip-multi-breaking, a recently reported droplet breakup mode^44^,^45^. Despite their non-uniformity, the satellites depend on oscillation, which may provide an additional handle to harness the formation of droplets with various volumes, a required feature in some applications. For example, in multi-volume droplet digital polymerase chain reaction (MV-dPCR)^46^, droplets with various volumes enable simultaneous measurements of a sample at different copies per droplet. Compared to single-volume digital PCR, MV-dPCR achieves higher detection reproducibility, wider dynamic range and better resolution while reducing the total number of droplets/wells required for the measurements^46^,^47^,^48^.

## Concluding Remarks

In conclusion, we have experimentally examined the dynamics of pinch-off in viscous two-fluid systems with oscillatory velocity actuated by mechanical perturbation. Attributed to the oscillatory flow, an enhanced suction of inner fluid towards the nozzle occurs in the last stage of pinch-off. In this scenario, the inner fluid thread thins radially faster prior to breakup compared to the unactuated case, still in a linear way though. The enhancement of pinch-off by external actuation depends on the oscillation amplitude. We rescale the actuated neck radius by a dimensionless pre-factor 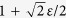, where *ε* is the dimensionless amplitude. Actuating the fluid system modulates the local force balance via increasing the effects of inner fluid inertia. Therefore, the enhanced fluid inertia is non-negligible and responsible for the accelerated neck thinning. Meanwhile, the actuated pinch-off displays a “less viscous” behavior due to the counterbalance between the enhanced inertia and the inner viscous resistance. Such a “less viscous” performance would enable robust control over breaking viscous liquid filament more easily. Moreover, the enhanced inner fluid inertia shifts the pinch-off location upstream towards the nozzle, which afterwards affects satellite droplet formation. By quantifying the relationship between the oscillation amplitude and the pinch-off location, novel control over satellites formation by oscillation could probably be developed.

Beyond technical benefit in modulating droplet formation resulted from our study, this work raises several questions of hydrodynamic interest but remaining to be addressed. Among them, two issues are of the most importance. One is that if the oscillation effect could dominate capillary effects, a new regime might occur, which may be similar to the thermal fluctuation dominant regime^20^,^21^. Another is that the existence of transient regimes during two-fluid pinch-off from the initial to final regime remains unexplored. Deeper understanding towards these issues calls for experimental studies in association with numerical simulations and theoretical explanations.

## Additional Information

**How to cite this article**: Zhu, P. *et al*. Pinch-off of microfluidic droplets with oscillatory velocity of inner phase flow. *Sci. Rep.* **6**, 31436; doi: 10.1038/srep31436 (2016).

## Supplementary Material

Supplementary Information

Supplementary Movie S1

Supplementary Movie S2

Supplementary Movie S3

Supplementary Movie S4

Supplementary Movie S5

Supplementary Movie S6

## References

[b1] Marieb E. N. & Hoehn K. Human Anatomy & Physiology. (Pearson Education, 2007).

[b2] Roy D. Communication signals and sexual selection in amphibians. Curr. Sci. 72, 923–927 (1997).

[b3] Hills R. L. Power from Steam: A History of the Stationary Steam Engine. (Cambridge University Press, 1993).

[b4] Ferrara K., Pollard R. & Borden M. Ultrasound microbubble contrast agents: fundamentals and application to gene and drug delivery. Annu. Rev. Biomed. Eng. 9, 415–447 (2007). 17651012 10.1146/annurev.bioeng.8.061505.095852

[b5] Collins D. J., Alan T., Helmerson K. & Neild A. Surface acoustic waves for on-demand production of picoliter droplets and particle encapsulation. Lab Chip 13, 3225–3231 (2013). 23784263 10.1039/c3lc50372k

[b6] Sauret A. & Shum H. C. Forced generation of simple and double emulsions in all-aqueous systems. Appl. Phys. Lett. 100, 154106 (2012).

[b7] Ziemecka I. . Monodisperse hydrogel microspheres by forced droplet formation in aqueous two-phase systems. Lab Chip 11, 620–624 (2011). 21125099 10.1039/c0lc00375a

[b8] Zhu P. A., Tang X. & Wang L. Q. Droplet generation in co-flow microfluidic channels with vibration. Microfluid. Nanofluid. 20, 47, 10.1007/s10404-016-1717-2 (2016).

[b9] Kong T. T., Liu Z., Song Y., Wang L. Q. & Shum H. C. Engineering polymeric composite particles by emulsion-templating: thermodynamics versus kinetics. Soft Matter 9, 9780–9784 (2013).

[b10] deMello A. J. Control and detection of chemical reactions in microfluidic systems. Nature 442, 394–402 (2006). 16871207 10.1038/nature05062

[b11] Kim S. H., Shim J. W. & Yang S. M. Microfluidic multicolor encoding of microspheres with nanoscopic surface complexity for multiplex immunoassays. Angew. Chem. Int. Ed. 50, 1171–1174 (2011). 10.1002/anie.201004869 21268220

[b12] Dittrich P. S. & Manz A. Lab-on-a-chip: microfluidics in drug discovery. Nat. Rev. Drug Discov. 5, 210–218 (2006). 16518374 10.1038/nrd1985

[b13] Mazzitelli S., Capretto L., Quinci F., Piva R. & Nastruzzi C. Preparation of cell-encapsulation devices in confined microenvironment. Adv. Drug Deliver. Rev. 65, 1533–1555 (2013). 10.1016/j.addr.2013.07.021 23933618

[b14] Eggers J. & Villermaux E. Physics of liquid jets. Rep. Prog. Phys. 71, 036601 (2008).

[b15] Lister J. R. & Stone H. A. Capillary breakup of a viscous thread surrounded by another viscous fluid. Phys. Fluids 10, 2758–2764 (1998).

[b16] Cohen I., Brenner M. P., Eggers J. & Nagel S. R. Two fluid drop snap-off problem: Experiments and theory. Phys. Rev. Lett. 83, 1147 (1999).

[b17] Zhang W. W. & Lister J. R. Similarity solutions for capillary pinch-off in fluids of differing viscosity. Phys. Rev. Lett. 83, 1151 (1999).

[b18] Cohen I. & Nagel S. R. Testing for scaling behavior dependence on geometrical and fluid parameters in the two fluid drop snap-off problem. Phys. Fluids 13, 3533–3541 (2001).

[b19] Sierou A. & Lister J. R. Self-similar solutions for viscous capillary pinch-off. J. Fluid Mech. 497, 381–403 (2003).

[b20] Hennequin Y. . Drop formation by thermal fluctuations at an ultralow surface tension. Phys. Rev. Lett. 97, 244502 (2006). 10.1103/PhysRevLett.97.244502 17280292

[b21] Petit J., Rivière D., Kellay H. & Delville J.-P. Break-up dynamics of fluctuating liquid threads. Proc. Natl. Acad. Sci. USA 109, 18327–18331 (2012). 10.1073/pnas.1207634109 PMC3494890 23090994

[b22] Eggers J. Dynamics of liquid nanojets. Phys. Rev. Lett. 89, 084502 (2002). 10.1103/PhysRevLett.89.084502 12190472

[b23] Louvet N., Bonn D. & Kellay H. Nonuniversality in the Pinch-Off of Yield Stress Fluids: Role of Nonlocal Rheology. Phys. Rev. Lett. 113, 218302 (2014). 10.1103/PhysRevLett.113.218302 25479525

[b24] Savage J. R., Caggioni M., Spicer P. T. & Cohen I. Partial universality: pinch-off dynamics in fluids with smectic liquid crystalline order. Soft Matter 6, 892–895 (2010).

[b25] Doshi P. . Persistence of memory in drop breakup: The breakdown of universality. Science 302, 1185–1188 (2003). 10.1126/science.1089272 14615531

[b26] Keim N. C., Møller P., Zhang W. W. & Nagel S. R. Breakup of air bubbles in water: Memory and breakdown of cylindrical symmetry. Phys. Rev. Lett. 97, 144503 (2006). 10.1103/PhysRevLett.97.144503 17155257

[b27] McKinley G. H. & Sridhar T. Filament-stretching rheometry of complex fluids. Annu. Rev. Fluid Mech. 34, 375–415 (2002).

[b28] Yildirim O. E. & Basaran O. A. Deformation and breakup of stretching bridges of Newtonian and shear-thinning liquids: comparison of one-and two-dimensional models. Chem. Eng. Sci. 56, 211–233 (2001).

[b29] Marmottant P. & Villermaux E. Fragmentation of stretched liquid ligaments. Phys. Fluids 16, 2732–2741 (2004).

[b30] Chen Y.-J. & Steen P. Dynamics of inviscid capillary breakup: collapse and pinchoff of a film bridge. J. Fluid Mech. 341, 245–267 (1997).

[b31] Garstecki P., Stone H. A. & Whitesides G. M. Mechanism for flow-rate controlled breakup in confined geometries: a route to monodisperse emulsions. Phys. Rev. Lett. 94, 164501 (2005). 10.1103/PhysRevLett.94.164501 15904231

[b32] Dollet B., van Hoeve W., Raven J.-P., Marmottant P. & Versluis M. Role of the Channel Geometry on the Bubble Pinch-Off in Flow-Focusing Devices. Phys. Rev. Lett. 100, 034504 (2008). 10.1103/PhysRevLett.100.034504 18232987

[b33] Fu T., Wu Y., Ma Y. & Li H. Z. Droplet formation and breakup dynamics in microfluidic flow-focusing devices: from dripping to jetting. Chem. Eng. Sci. 84, 207–217 (2012).

[b34] Du W., Fu T., Zhu C., Ma Y. & Li H. Z. Breakup dynamics for high-viscosity droplet formation in a flow‐focusing device: Symmetrical and asymmetrical ruptures. AICHE J. 62, 325–337 (2016).

[b35] Meier G., Klöpper A. & Grabitz G. The influence of kinematic waves on jet break down. Exp. Fluids 12, 173–180 (1992).

[b36] Sauret A., Spandagos C. & Shum H. C. Fluctuation-induced dynamics of multiphase liquid jets with ultra-low interfacial tension. Lab Chip 12, 3380–3386 (2012). 10.1039/c2lc40524e 22773244

[b37] Batchelor G. An Introduction to Fluid Dynamics. (Cambridge University Press, 1967).

[b38] Tomotika S. On the instability of a cylindrical thread of a viscous liquid surrounded by another viscous fluid. Proc. R. Soc. Lond. A 150, 322–337 (1935).

[b39] Castrejón-Pita J. R. . Plethora of transitions during breakup of liquid filaments. Proc. Natl. Acad. Sci. USA 112, 4582–4587 (2015). 10.1073/pnas.1418541112 PMC4403208 25825761

[b40] Eggers J. Nonlinear dynamics and breakup of free-surface flows. Rev. Mod. Phys. 69, 865–929 (1997).

[b41] Chaudhary K. & Maxworthy T. The nonlinear capillary instability of a liquid jet. Part 3. Experiments on satellite drop formation and control. J. Fluid Mech. 96, 287–297 (1980).

[b42] Tjahjadi M., Stone H. & Ottino J. Satellite and subsatellite formation in capillary breakup. J. Fluid Mech. 243, 297–317 (1992).

[b43] Stone H. A. Dynamics of drop deformation and breakup in viscous fluids. Annu. Rev. Fluid Mech. 26, 65–102 (1994).

[b44] Zhu P. A., Kong T. T., Kang Z. X., Tian X. W. & Wang L. Q. Tip-multi-breaking in capillary microfluidic devices. Sci. Rep. 5, 10.1038/srep11102 (2015). PMC4468424 26077155

[b45] Zhu P. A. . Droplet breakup in expansion-contraction microchannels. Sci. Rep. 6, 10.1038/srep21527 (2016). PMC4761913 26899018

[b46] Zeng Y., Shin M. & Wang T. Programmable active droplet generation enabled by integrated pneumatic micropumps. Lab Chip 13, 267–273 (2013). 10.1039/c2lc40906b 23160148

[b47] Kreutz J. E. . Theoretical design and analysis of multivolume digital assays with wide dynamic range validated experimentally with microfluidic digital PCR. Anal. Chem. 83, 8158–8168 (2011). 10.1021/ac201658s PMC3216679 21981344

[b48] Shen F. . Multiplexed quantification of nucleic acids with large dynamic range using multivolume digital RT-PCR on a rotational SlipChip tested with HIV and hepatitis C viral load. J. Am. Chem. Soc. 133, 17705–17712 (2011). 10.1021/ja2060116 PMC3216675 21995644

