# Supplementary material for: Pinch-off of microfluidic droplets with oscillatory velocity of inner phase flow
Source: Sci Rep. 2016 Aug 11;6:31436. doi: 10.1038/srep31436 (PMC4980598; doi:10.1038/srep31436)
Supplement: Supplementary Information [file srep31436-s1.pdf]

Supplementary Information

"Pinch-off of microfluidic droplets with oscillatory velocity"

Pingan Zhu[1,2], Xin Tang[1,2], Ye Tian[1,2] and Liqiu Wang[1,2,] *

[1]Department of Mechanical Engineering, the University of Hong Kong, Hong Kong

[2]HKU-Zhejiang Institute of Research and Innovation (HKU-ZIRI), 311300, Hangzhou, Zhejiang, China

* Corresponding author: lqwang@hku.hk

This file includes:

1. Legends of Supplementary Movies S1 to S6
2. Validation of kR_0 >1 when $f \geq 100$ Hz
3. Derivation of the Linear Scaling of Neck Diameter
4. Estimate of $F(\lambda)$ Using Linear Stability Analysis
5. Supplementary Figures

1. Legends of Supplementary Movies S1 to S5

1.1 Supplementary Movie S1: Unperturbed Two-fluid Drop Pinch-off

Droplet pinch-off without external perturbation. The final stage of pinch-off is governed by Stokes flow, and inner liquid neck-thinning rate is a constant that only depends on material properties. Asymmetric pinch-off indicates that front pinch-off occurs before rear pinch-off. Five visible satellite droplets are generated.

1.2 Supplementary Movie S2: Perturbed Two-fluid Drop Pinch-off

Droplet pinch-off perturbed with amplitude $\varepsilon_0 = 1\,\text{mm}$. Symmetric necks at front and rear sites are formed simultaneously. Five visible satellite droplets are generated.

1.3 Supplementary Movie S3: Perturbed Two-fluid Drop Pinch-off

Droplet pinch-off perturbed with amplitude $\varepsilon_0 = 2\,\text{mm}$. Asymmetric necks are formed with rear pinch-off occurring before front pinch-off. Six visible satellite droplets are generated.

1.4 Supplementary Movie S4: Perturbed Two-fluid Drop Pinch-off

Droplet pinch-off perturbed with amplitude $\varepsilon_0 = 3\,\text{mm}$. Rear pinch-off occurs first, followed by the front pinch-off. The first pinch-off takes place at the ejection nozzle. Six visible satellite droplets are generated.

1.5 Supplementary Movie S5: Perturbed Two-fluid Drop Pinch-off

Droplet pinch-off perturbed with amplitude $\varepsilon_0 = 5\,\text{mm}$. Rear pinch-off occurs before front pinch-off. The rear pinch-off takes place backwards inside the nozzle. Ten visible satellite droplets are generated.

1.6 Supplementary Movie S6: Identical Dynamics in Adjacent Perturbed Pinch-off

This movie displays droplet growth and pinch-off subjected to perturbation of amplitude $\varepsilon_0 = 3\,\text{mm}$ and frequency f = 12 Hz. Every perturbed pinch-off is identical to each other. In this movie, 70 wt.% glycerol + 30 wt.% water was inner fluid, and silicone oil was outer fluid with viscosity ratio being 0.0387. The movie was recoded at 5,000 frame per second (fps), and played at 500 fps for droplet growth and 50 fps for droplet pinch-off to emphasize the pinch-off dynamics.

For Movies S1-S5, the neck-thinning rate for perturbed case is accelerated by the perturbation amplitude ε_0, but still constant prior to pinch-off. All movies were captured at the speed of 6,820 fps, and played at 341 fps. Liquids in the exampled movies were 84 wt.% glycerol + 16 wt.% water as inner fluid, and silicone oil as outer fluid. The viscosity ratio of this two-fluid system is 0.157. For perturbed pinch-off, perturbation frequency was kept as a constant of 26 Hz.

2. Validation of kR_0 >1 when $f \geq 100$ Hz

For the experimental setup used, R_0 is evaluated to be $R_0 = 0.5D_n$, where D_n = 151.443 μm, the outer diameter of the ejection nozzle. The wave number k is related to wave length λ_w to be:

$$k = 2\pi / \lambda_w . \tag{A1}$$

We also have

$$\lambda_w f = V_0 . \tag{A2}$$

where $V_0 = 4Q_i / \pi D_n^2$ is the average ejection velocity of inner fluid. Combining Eqs. (A1) and (A2), we get the formula for k as follows:

$$k = \pi^2 D_n^2 f / 2Q_i . \tag{A3}$$

As such, kR_0 is given by Eq. (A4):

$$kR_0 = \pi^2 D_n^3 f / 4Q_i . \tag{A4}$$

In our experiments, 0.02 mL $h^{-1} \leq Q_i \leq$ 0.45 mL h^{-1}, so that $kR_0 \geq kR_0$ (Q_i = 0.45 mL h^{-1}, f = 100 Hz) when $f \geq$ 100 Hz. A simple calculation gives kR_0 (Q_i = 0.45 mL h^{-1}, f = 100 Hz) = 6.856 > 1. This validates that when $f \geq$ 100 Hz, kR_0 > 1 for our experimental setup.

3. Derivation of the Linear Scaling of Neck Diameter

In the viscous-capillary force balance analysis, inner axial viscous stress ($\sim \eta_i u / z$, with $u \sim z / \tau$), outer radial viscous stress ($\sim \eta_o u / R_{\min}$), and capillary pressure ($\sim \gamma / R_{\min}$) compete with each other. Firstly, $\eta_i u / z \sim \eta_o u / R_{\min}$ simply gives $R_{\min} \sim z$, so that $R_{\min} = g(\lambda) z$ with $\lambda = \eta_i / \eta_o$ and $g(\lambda)$ being a function of λ. Then $\eta_o u / R_{\min} \sim \gamma / R_{\min}$ leads to $u \sim \gamma / \eta_o$. Since $u \sim z / \tau$, z then scales as $z \sim \gamma\tau / \eta_o$. Given that $R_{\min} = g(\lambda) z$, $R_{\min}$ can be written in the form of $R_{\min} = f(\lambda)\gamma\tau / \eta_o$, with $f(\lambda)$ being a function of λ. Replacing $R_{\min}$ by $D_{\min}$, one finally gets the scaling of neck diameter, $D_{\min} = F(\lambda)\gamma\tau / \eta_o$, with $F(\lambda)$ being evaluated by linear stability analysis.

4. Estimate of $F(\lambda)$ Using Linear Stability Analysis

Here we consider the case in which an initial infinitesimal perturbation grows on a long cylindrical viscous jet with viscosity η_i immersed in another viscous liquid with viscosity η_o, and the growing perturbation is proportional to $e^{i(\omega t+kz)}$. According to Tomotika [1], the dispersion relation gives,

$$i\omega = \frac{\gamma}{2R_0\eta_o}(1-x^2)\Phi(x,\lambda)\,, \tag{A5}$$

where ω is frequency of the perturbation, γ the interfacial tension, and R_0 the radius of the unperturbed jet. $x = kR_0$ is the dimensionless wave number, where $k = 2\pi / l$ is the wave number with l being the wavelength of the perturbation. $\Phi(x,\lambda)$ is a function of x and viscosity ratio λ in the form of

$$\Phi(x,\lambda) = \frac{N(x,\lambda)}{D(x,\lambda)}, \tag{A6}$$

with

$$N(x,\lambda) = I_1(x)\Delta_1 - \left[xI_0(x) - I_1(x)\right]\Delta_2\,, \tag{A7}$$

$$\begin{aligned} D(x,\lambda) = {} & \lambda\left[xI_0(x)-I_1(x)\right]\Delta_1 - \lambda\left[(x^2+1)I_1(x)-xI_0(x)\right]\Delta_2 \\ & -\left[xK_0(x)+K_1(x)\right]\Delta_3 - \left[(x^2+1)K_1(x)+xK_0(x)\right]\Delta_4 \end{aligned}\,. \tag{A8}$$

In Eqs. (A7) and (A8), $I(x)$ and $K(x)$ are the modified Bessel functions, with subscripts “0” and “1” representing the order. In addition, Δ_1, Δ_2, Δ_3 and Δ_4 are functions of x and λ, given in the following form:

$$\Delta_1 = \begin{vmatrix} xI_0(x)-I_1(x) & K_1(x) & -xK_0(x)-K_1(x) \\ I_0(x)+xI_1(x) & -K_0(x) & -K_0(x)+xK_1(x) \\ \lambda xI_0(x) & K_1(x) & -xK_0(x) \end{vmatrix}, \tag{A9}$$

$$\Delta_2 = \begin{vmatrix} I_1(x) & K_1(x) & -xK_0(x)-K_1(x) \\ I_0(x) & -K_0(x) & -K_0(x)+xK_1(x) \\ \lambda I_1(x) & K_1(x) & -xK_0(x) \end{vmatrix}, \tag{A10}$$

$$\Delta_3 = \begin{vmatrix} I_1(x) & xI_0(x) - I_1(x) & -xK_0(x) - K_1(x) \\ I_0(x) & I_0(x) + xI_1(x) & -K_0(x) + xK_1(x) \\ \lambda I_1(x) & \lambda x I_0(x) & -xK_0(x) \end{vmatrix}, \tag{A11}$$

$$\Delta_4 = \begin{vmatrix} I_1(x) & xI_0(x) - I_1(x) & K_1(x) \\ I_0(x) & I_0(x) + xI_1(x) & -K_0(x) \\ \lambda I_1(x) & \lambda x I_0(x) & K_1(x) \end{vmatrix}. \tag{A12}$$

Assuming that inner liquid neck-thinning rate cannot be faster than the fastest growing linear perturbation in Eq. (A5), we have [2-4]:

$$t^{-1} = \partial_t R_{\min} / R_{\min} < i\omega|_{\max}. \tag{A13}$$

Therefore, the upper bound for the evolution of dynamical neck diameter D_{min} gives,

$$D_{\min} < [(1-x^2)\Phi(x,\lambda)]|_{\max} \frac{\gamma}{\eta_{out}} t. \tag{A14}$$

Based on Eq. (A14), it is clear to extract $F(\lambda)$ as the maxima of $(1-x^2)\Phi(x,\lambda)$ in the domain of x, so that

$$F(\lambda) = [(1-x^2)\Phi(x,\lambda)]_{\max}. \tag{A15}$$

Take $\lambda = 0.039$ as example, $F(\lambda) = 0.3363$, as shown in Fig. S4. Finally, we present the value of $F(\lambda)$ as a function of λ in Fig. S5.

1 Tomotika, S. On the instability of a cylindrical thread of a viscous liquid surrounded by another viscous fluid. *Proc. R. Soc. Lond. A* **150**, 322-337 (1935).

2 Cohen, I., Brenner, M. P., Eggers, J. & Nagel, S. R. Two fluid drop snap-off problem: Experiments and theory. *Phys. Rev. Lett.* **83**, 1147 (1999).

3 Zhang, W. W. & Lister, J. R. Similarity solutions for capillary pinch-off in fluids of differing viscosity. *Phys. Rev. Lett.* **83**, 1151 (1999).

4 Cohen, I. & Nagel, S. R. Testing for scaling behavior dependence on geometrical and fluid parameters in the two fluid drop snap-off problem. *Phys. Fluids* **13**, 3533-3541 (2001).

5. Supplementary Figures

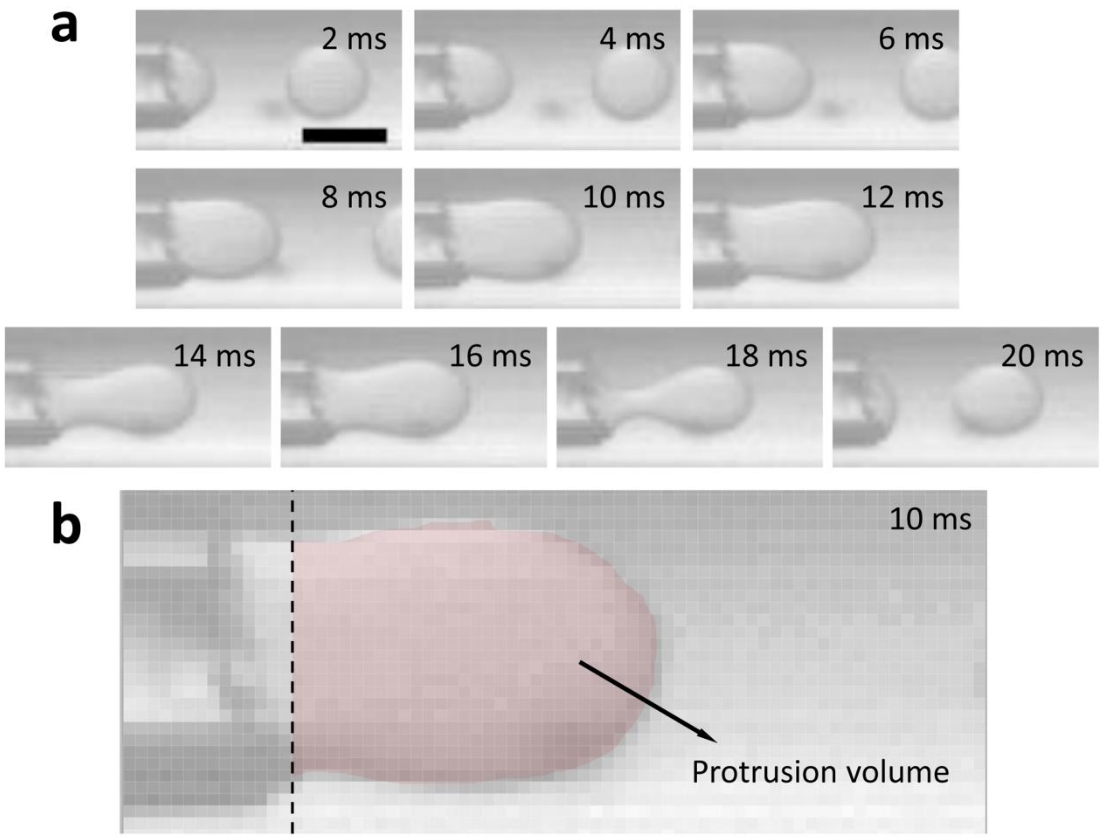

Figure S1 | Drop generation subjected to external vibration of ε_0 = 5 mm and f = 50 Hz. (**a**) Snapshots of a droplet production. The generation frequency of the droplet is synchronized with the vibration frequency of 50 Hz. Scale bar, 100 μm. (**b**) Magnification of the snapshot showing the transient volume (shadow region) of the protrusion during one droplet generation, exampled by time instant at 10 ms. The protrusion volume indicates how much inner fluid is transiently discharged, which is calculated in Matlab by using the binary image of the snapshot. Every square cell in **b** corresponds to one pixel of the image. Q_i = 0.1 mL h^{-1} and Q_o = 1 mL h^{-1}. Viscosity ratio, λ = 0.0387.

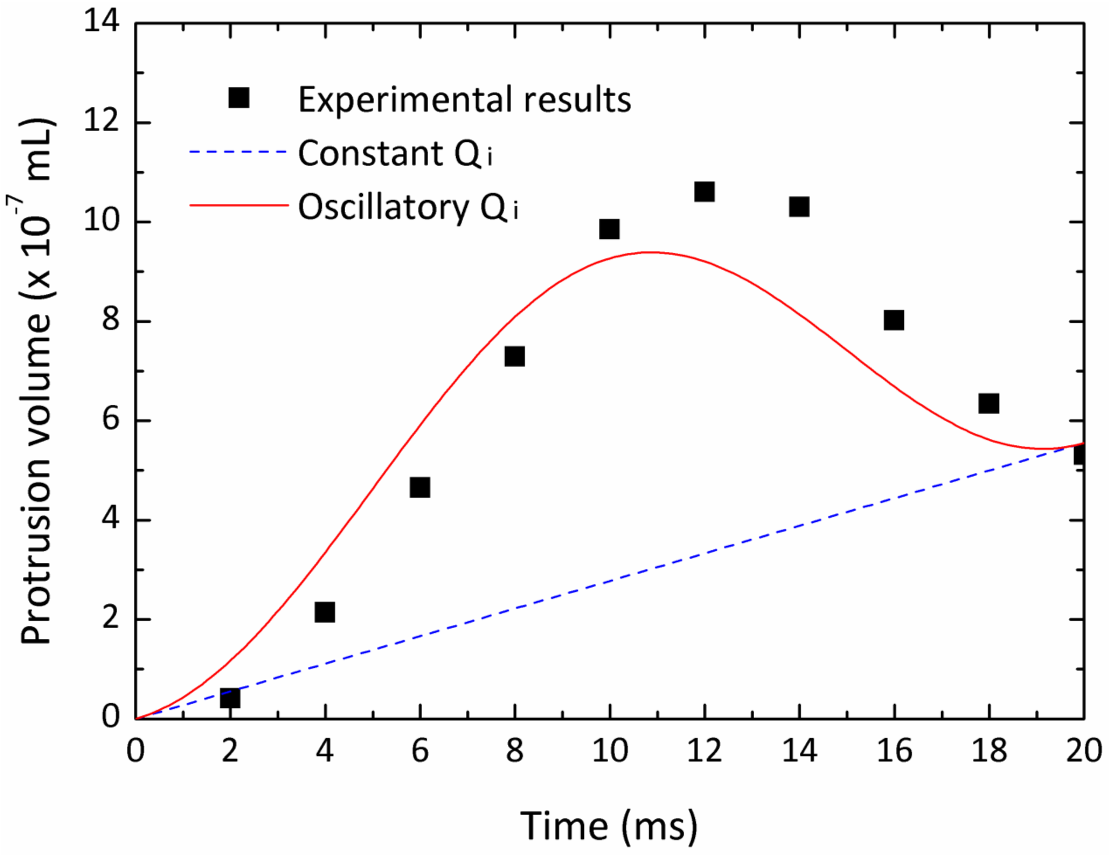

Figure S2 | Validation of Eq. (4). Experimental data are measured from Fig. S1. The dashed line represents the prediction from constant Q_i in the form of $V_{pro} = Q_i t$, with V_{pro} denoting the protrusion volume. The solid curve stands for the prediction of $V_{pro} = Q_i[t + \frac{\varepsilon}{2\pi f}(1 - \cos(2\pi f t))]$, obtained by integrating Eq. (4), where Q_i = 0.1 mL h^{-1}, ε = 3.67 and f = 50 Hz. The predictions of solid curve fairly agree with the experimental results, while the slight deviation might come from other effects that also contribute to the inner flow oscillation, such as the shaking of microtubing.

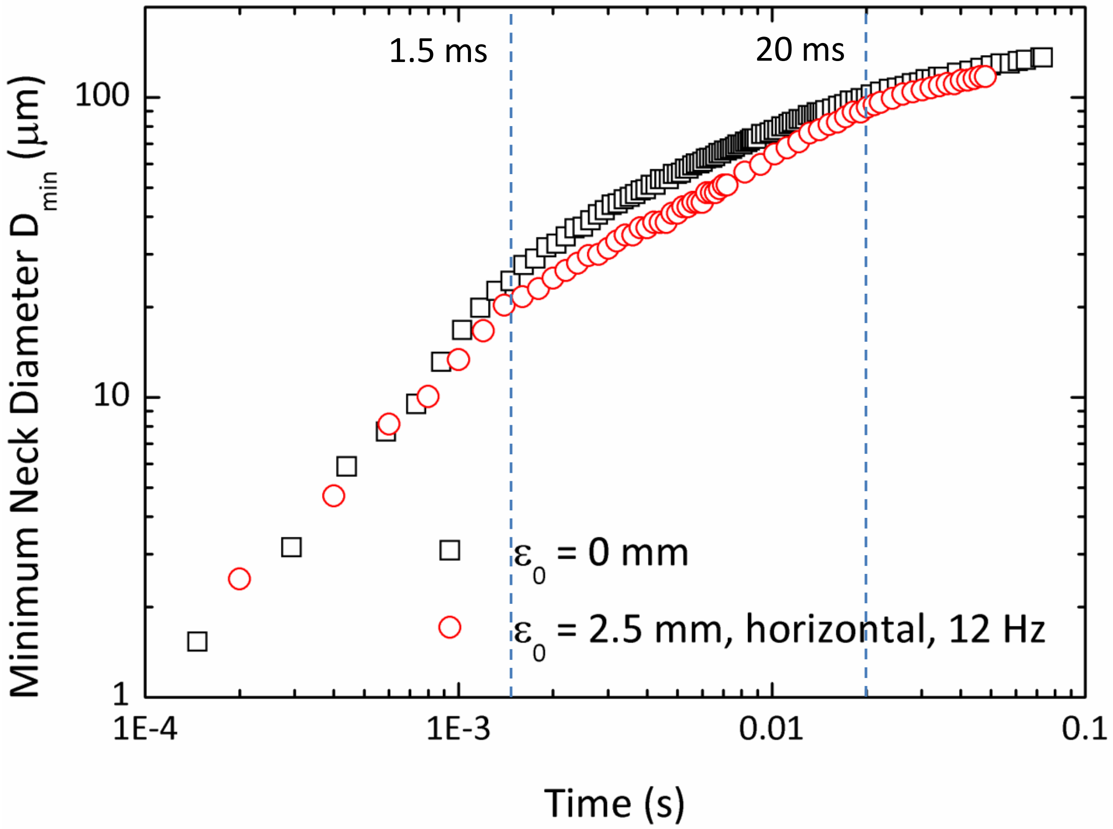

Figure S3 | Comparison of neck pinch-off with and without external perturbation in the direction of horizontal plane. Although the deviation in the pinch-off dynamics during ~ 1.5 – 20 ms may indicate the influence of factors except gravity on neck pinch-off, no apparent difference is observed in the vicinity of pinch-off (time t < 1.5 ms, Stokes regime). This figure, compared to Figs. 2-3 in the context, confirms that gravity is the dominant effect in enhancing neck pinch-off in Stokes regime. $Q_i = 0.45$ mL h^{-1}, $Q_o = 1$ mL h^{-1}. Viscosity ratio, $\lambda = 0.0387$.

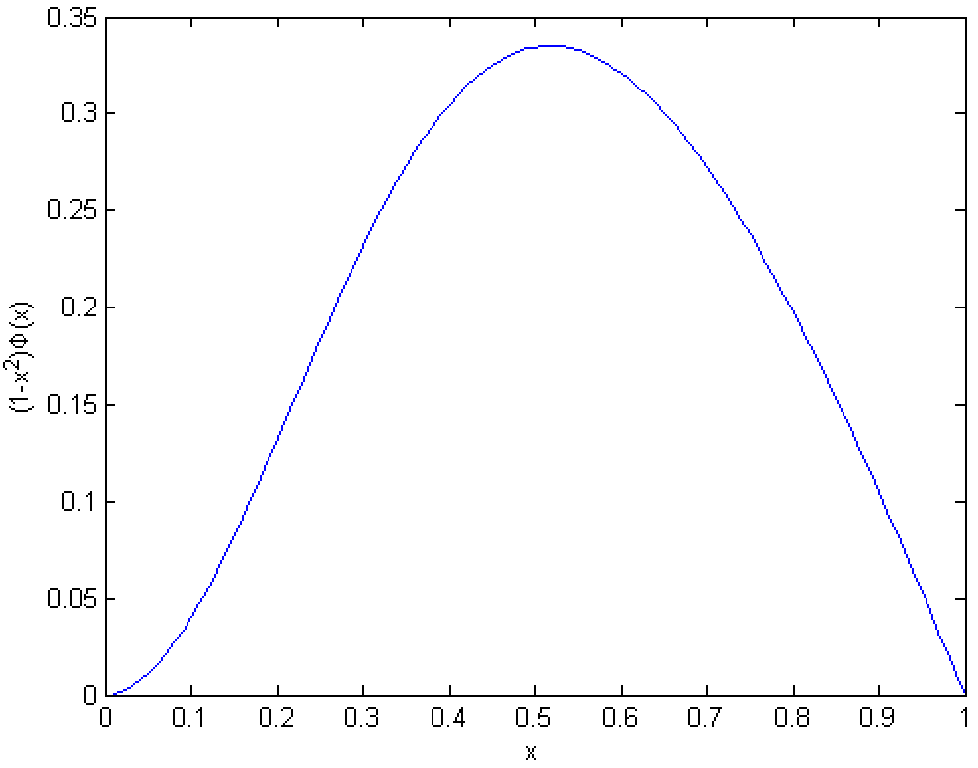

Figure S4 | (1-x^2)Φ(x, λ) as a function of x with viscosity ratio λ = 0.039. The maxima of $(1-x^2)\Phi(x,\lambda)$ corresponds to the value of $F(\lambda)$, which is 0.3363.

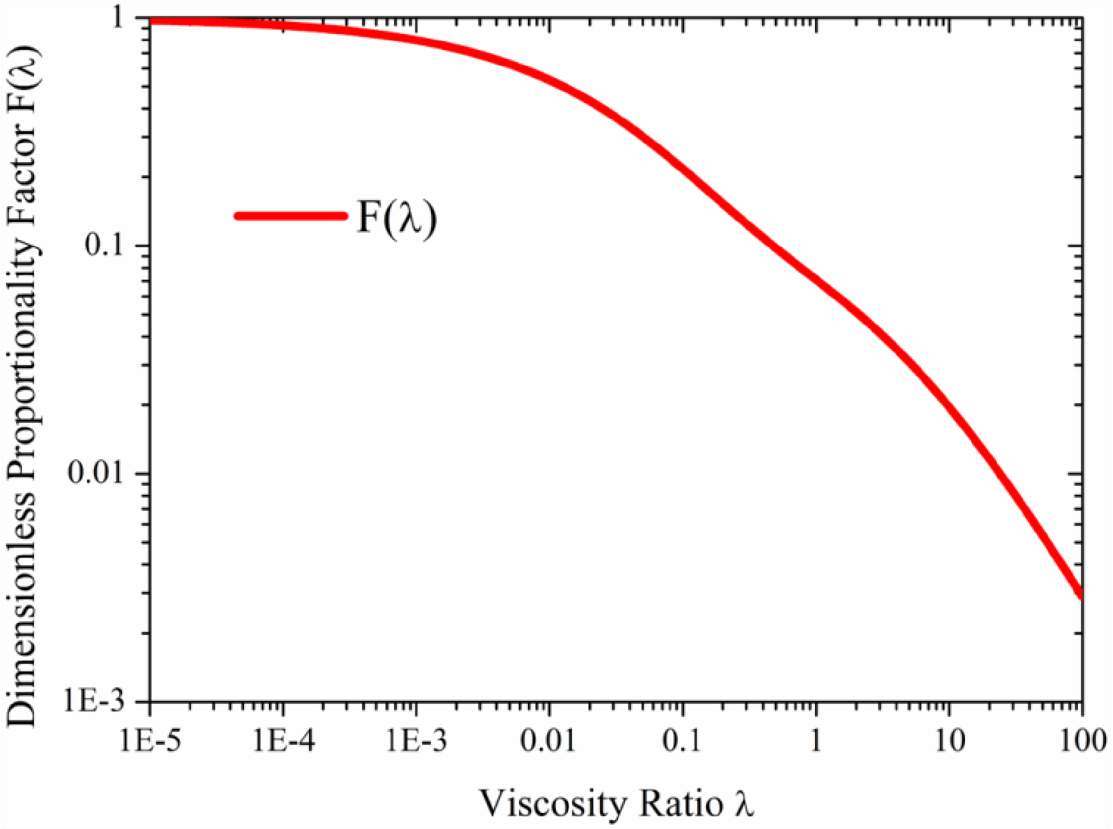

Figure S5 | *F*(*λ*) as a function of viscosity ratio *λ*.
